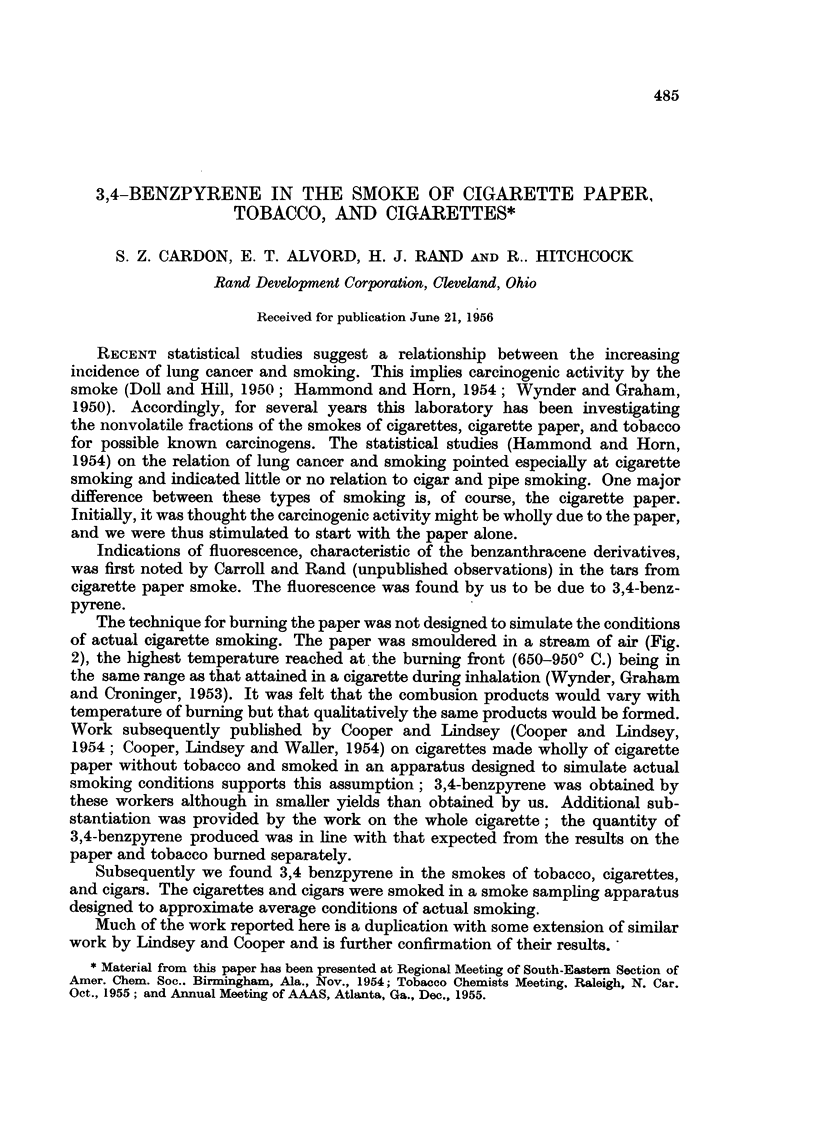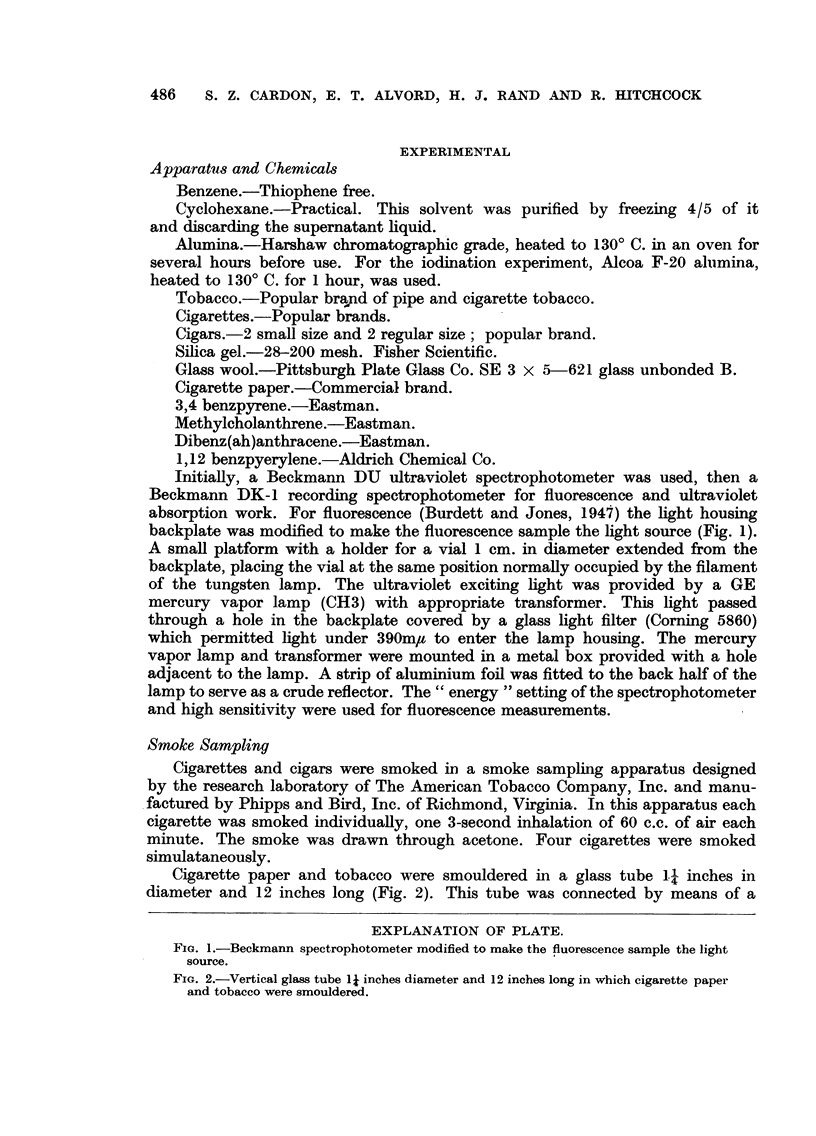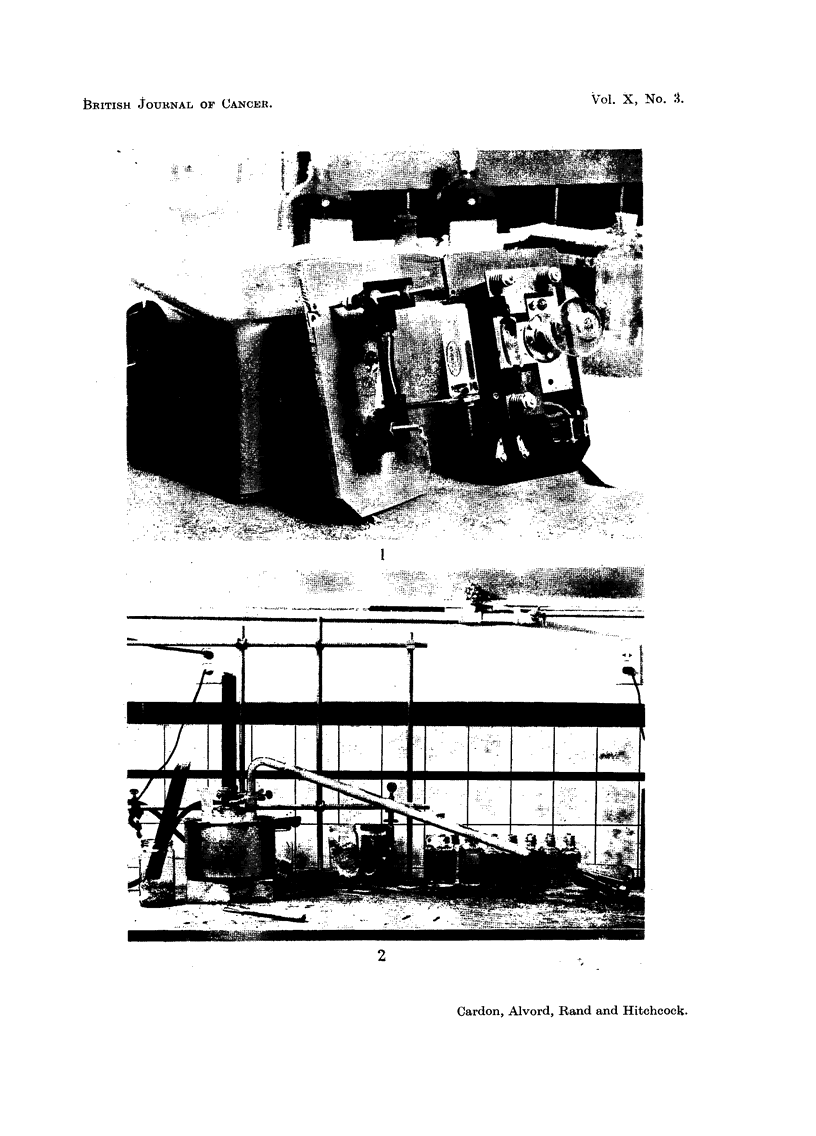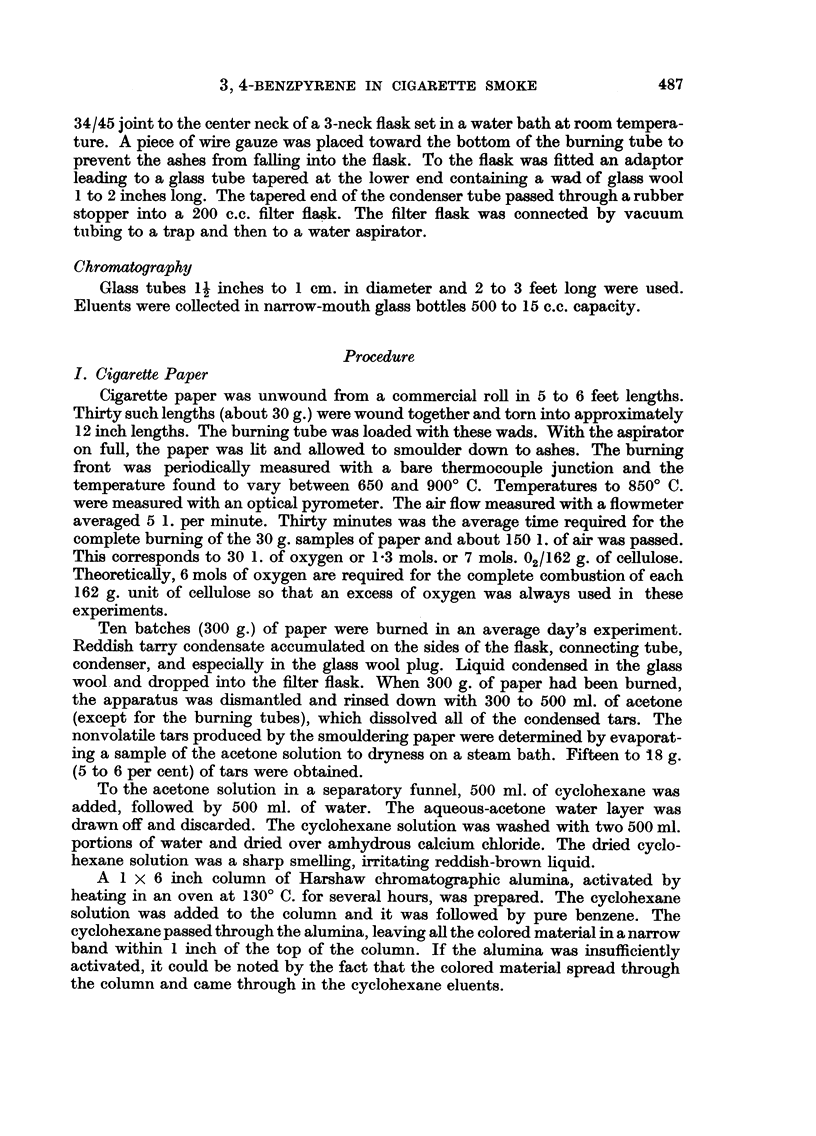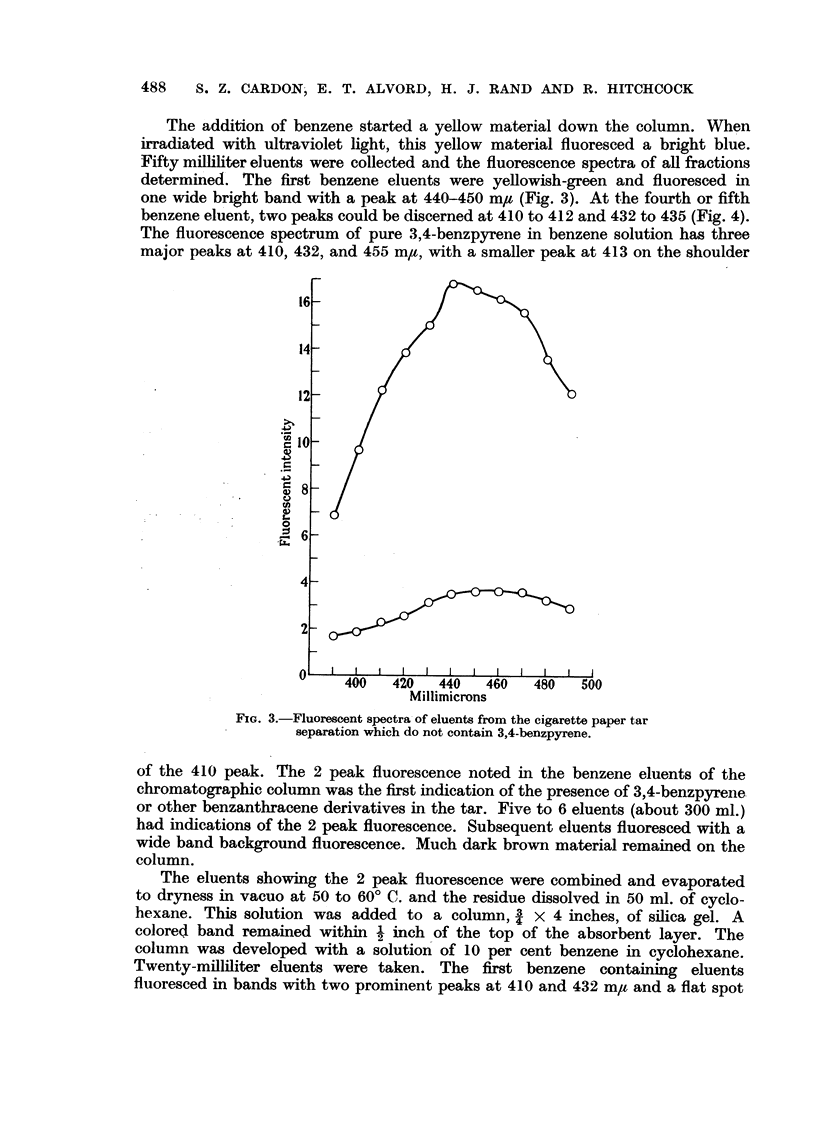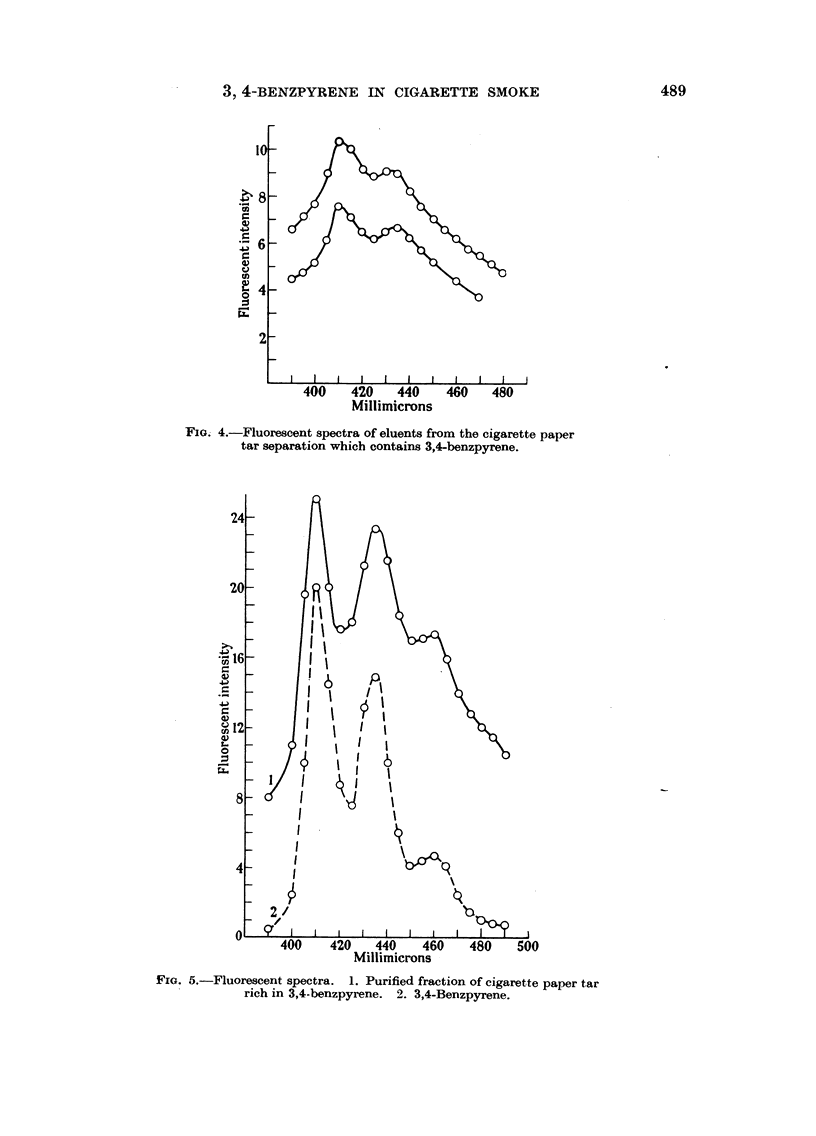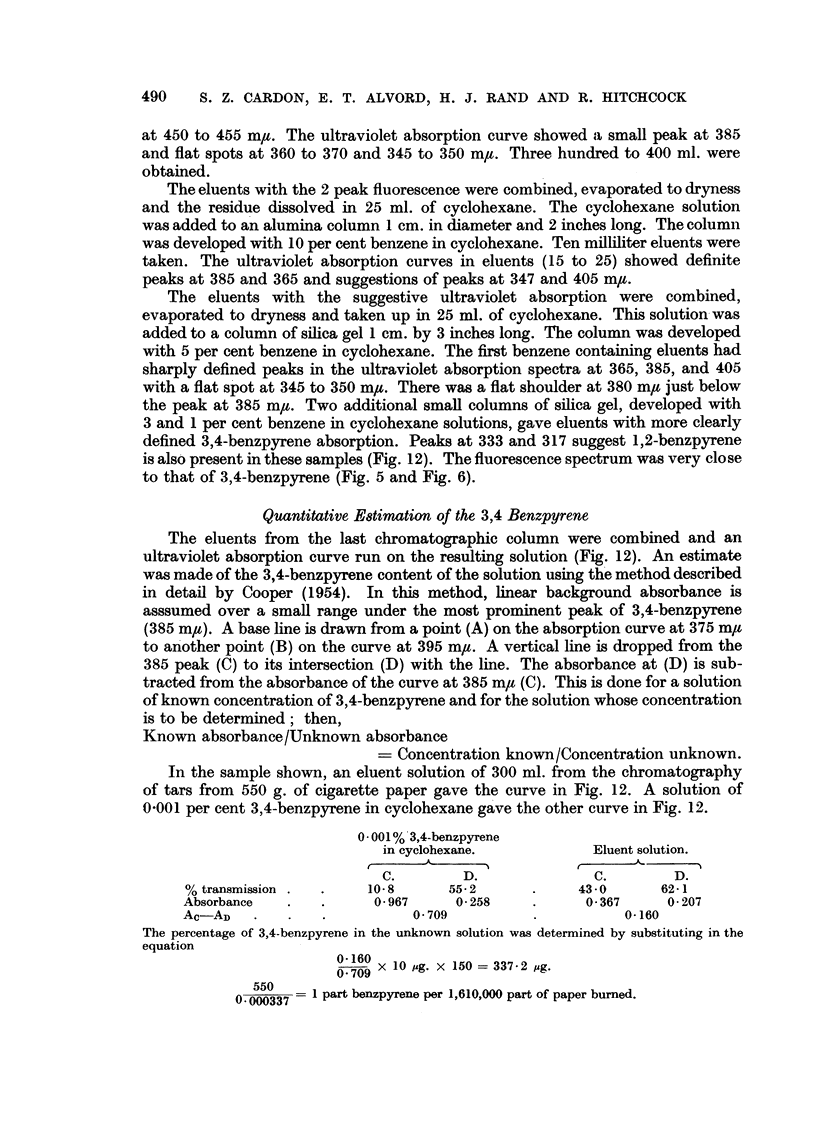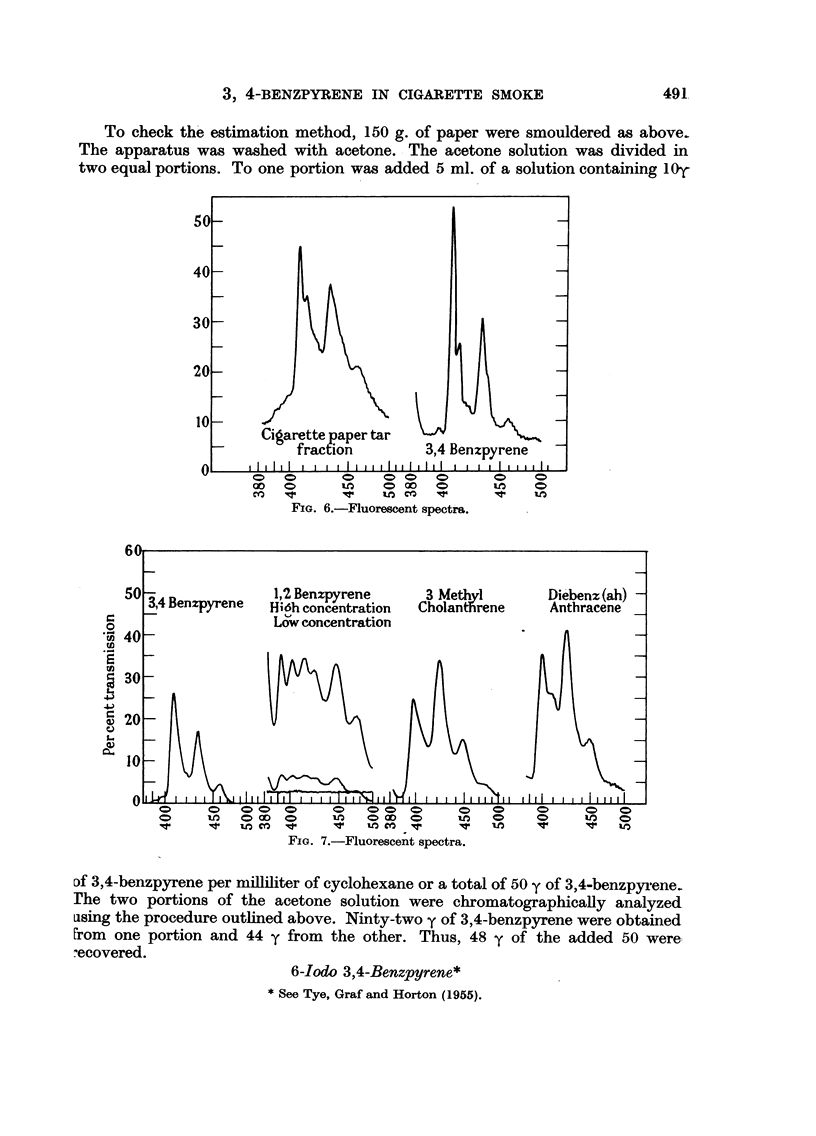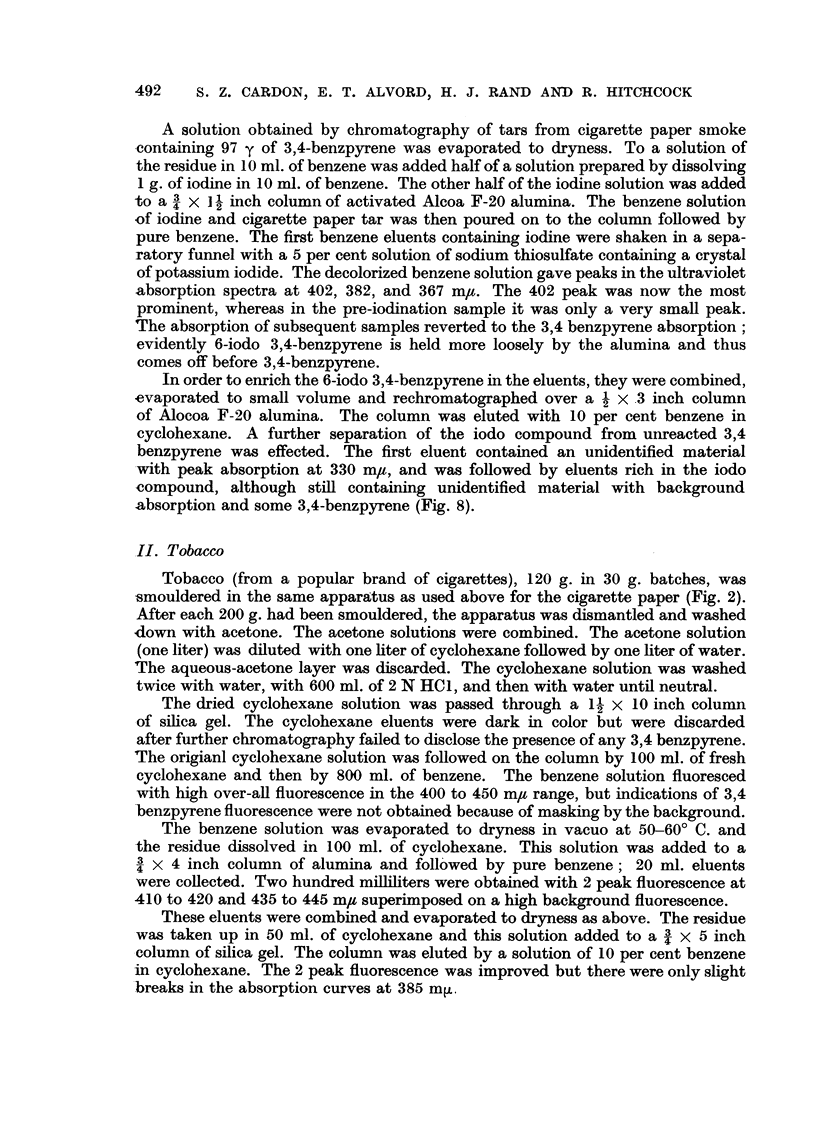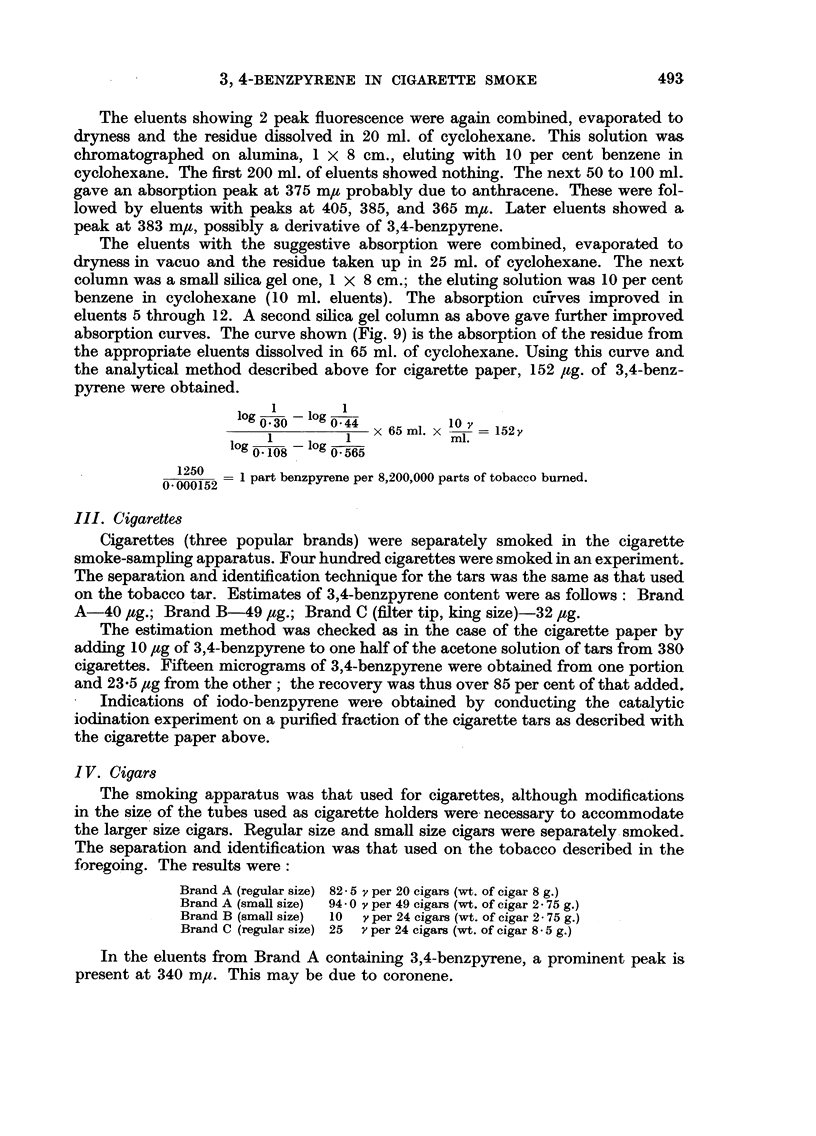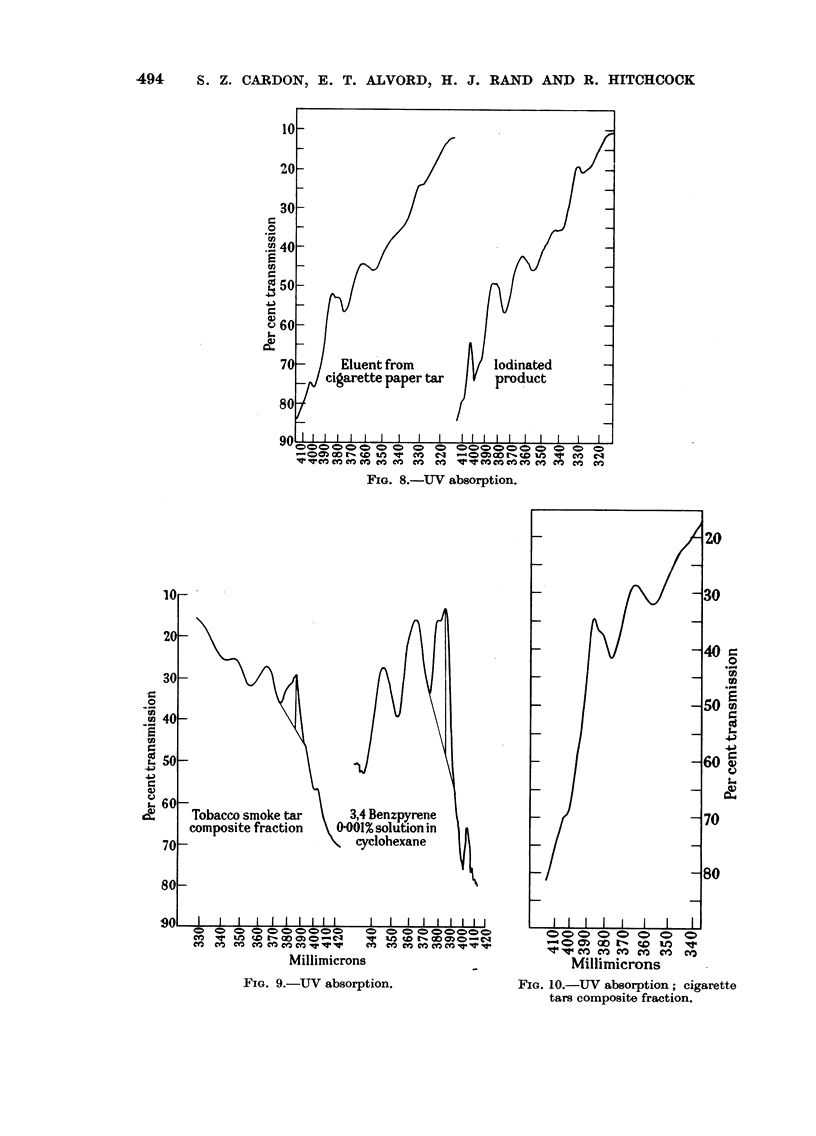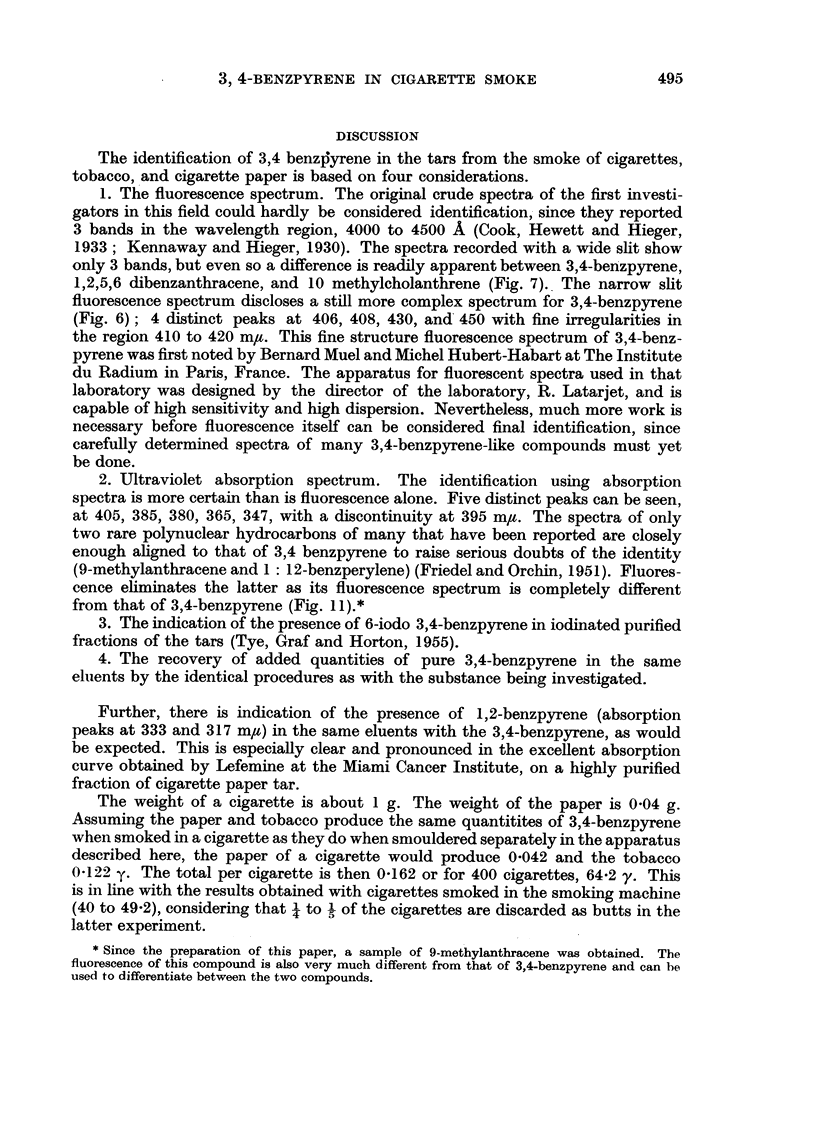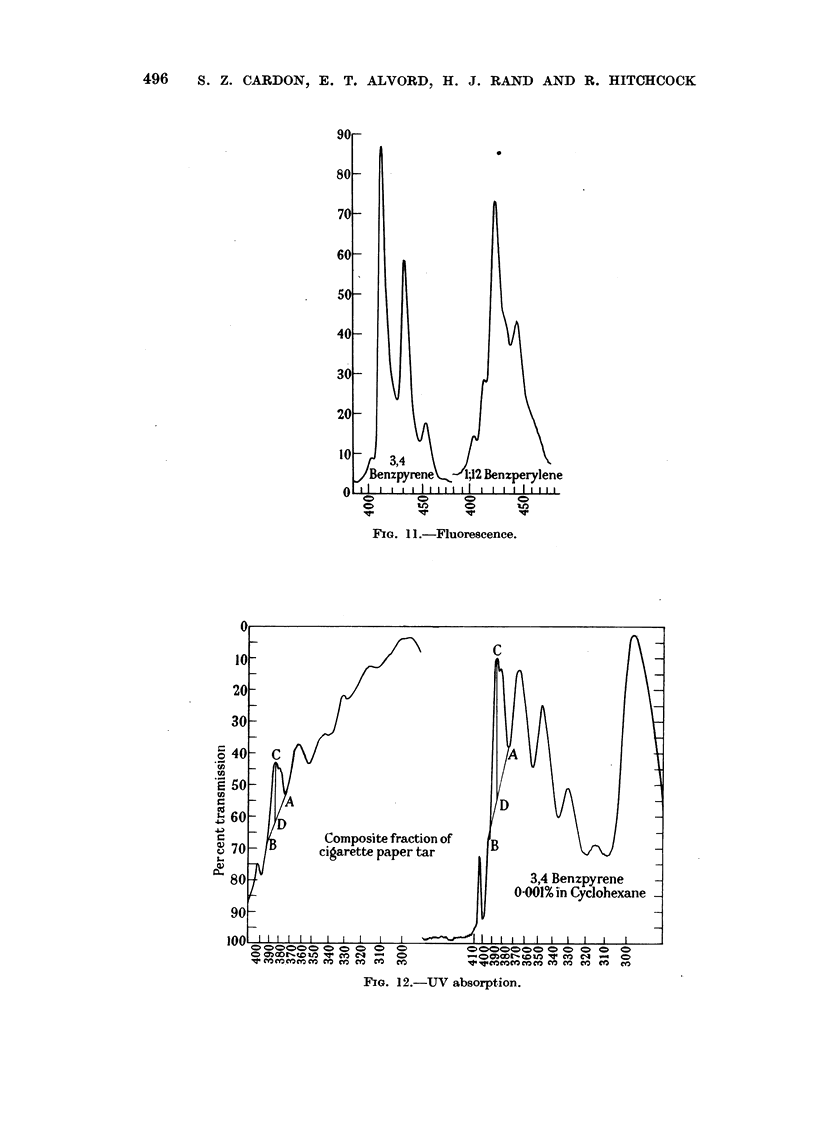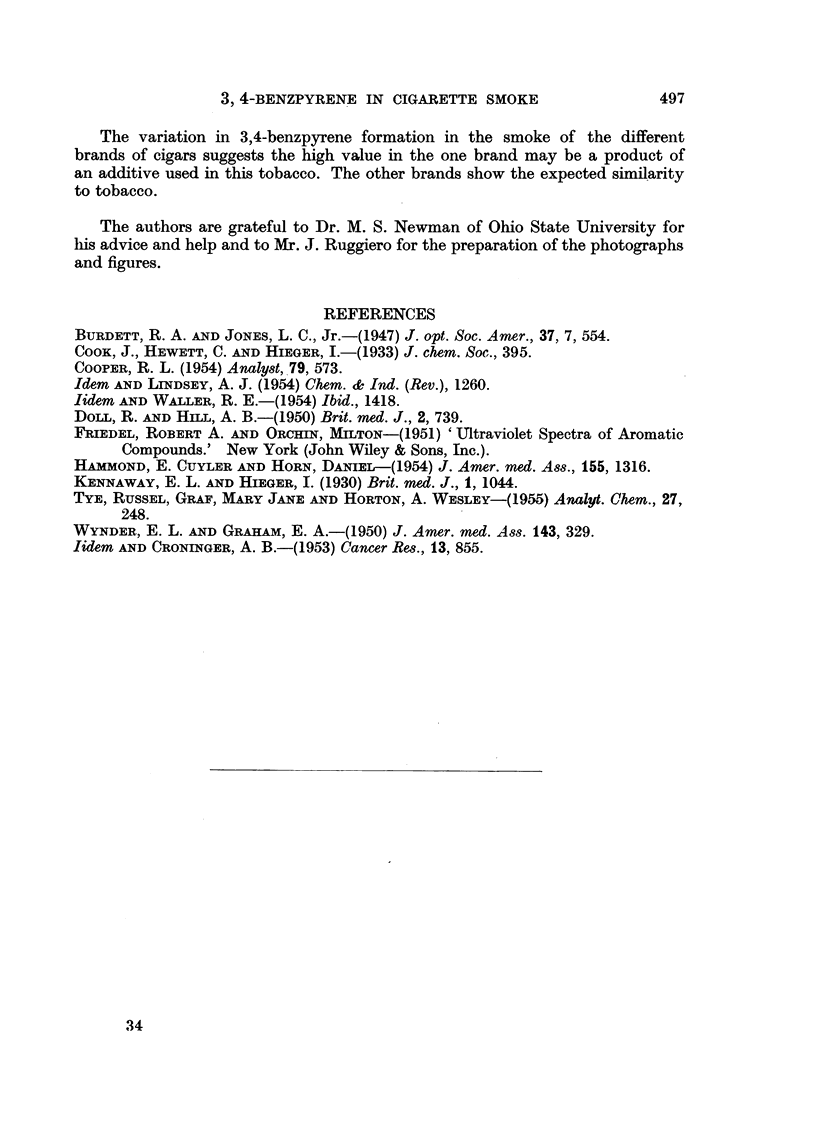# 3,4-Benzpyrene in the Smoke of Cigarette Paper, Tobacco, and Cigarettes*

**DOI:** 10.1038/bjc.1956.55

**Published:** 1956-09

**Authors:** S. Z. Cardon, E. T. Alvord, H. J. Rand, R. Hitchcock

## Abstract

**Images:**


### 3,4-BENZPYRENE IN THE SMOKE OF CIGARETTE PAPER, TOBACCO, AND CIGARETTES*

S. Z. CARDON, E. T. ALVORD, H. J. RAND AND R.. HITCHCOCK

*Rand Development Corporation, Cleveland, Ohio*

Received for publication June 21, 1956

RECENT statistical studies suggest a relationship between the increasing incidence of lung cancer and smoking. This implies carcinogenic activity by the smoke (Doll and Hill, 1950 ; Hammond and Horn, 1954 ; Wynder and Graham, 1950). Accordingly, for several years this laboratory has been investigating the nonvolatile fractions of the smokes of cigarettes, cigarette paper, and tobacco for possible known carcinogens. The statistical studies (Hammond and Horn, 1954) on the relation of lung cancer and smoking pointed especially at cigarette smoking and indicated little or no relation to cigar and pipe smoking. One major difference between these types of smoking is, of course, the cigarette paper. Initially, it was thought the carcinogenic activity might be wholly due to the paper, and we were thus stimulated to start with the paper alone.

Indications of fluorescence, characteristic of the benzanthracene derivatives, was first noted by Carroll and Rand (unpublished observations) in the tars from cigarette paper smoke. The fluorescence was found by us to be due to 3,4-benzpyrene.

The technique for burning the paper was not designed to simulate the conditions of actual cigarette smoking. The paper was smouldered in a stream of air (Fig. 2), the highest temperature reached at the burning front (650–950° C.) being in the same range as that attained in a cigarette during inhalation (Wynder, Graham and Croninger, 1953). It was felt that the combusion products would vary with temperature of burning but that qualitatively the same products would be formed. Work subsequently published by Cooper and Lindsey (Cooper and Lindsey, 1954 ; Cooper, Lindsey and Waller, 1954) on cigarettes made wholly of cigarette paper without tobacco and smoked in an apparatus designed to simulate actual smoking conditions supports this assumption ; 3,4-benzpyrene was obtained by these workers although in smaller yields than obtained by us. Additional substantiation was provided by the work on the whole cigarette ; the quantity of 3,4-benzpyrene produced was in line with that expected from the results on the paper and tobacco burned separately.

Subsequently we found 3,4 benzpyrene in the smokes of tobacco, cigarettes, and cigars. The cigarettes and cigars were smoked in a smoke sampling apparatus designed to approximate average conditions of actual smoking.

Much of the work reported here is a duplication with some extension of similar work by Lindsey and Cooper and is further confirmation of their results.

* Material from this paper has been presented at Regional Meeting of South-Eastern Section of Amer. Chem. Soc.. Birmingham, Ala., Nov., 1954; Tobacco Chemists Meeting, Raleigh, N. Car. Oct., 1955 ; and Annual Meeting of AAAS, Atlanta, Ga., Dec., 1955.

### EXPERIMENTAL

### *Apparatus and Chemicals*

Benzene.—Thiophene free.

Cyclohexane.—Practical. This solvent was purified by freezing 4/5 of it and discarding the supernatant liquid.

Alumina.—Harshaw chromatographic grade, heated to 130° C. in an oven for several hours before use. For the iodination experiment, Alcoa F-20 alumina, heated to 130° C. for 1 hour, was used.

Tobacco.—Popular brand of pipe and cigarette tobacco.

Cigarettes.—Popular brands.

Cigars.—2 small size and 2 regular size ; popular brand.

Silica gel.—28–200 mesh. Fisher Scientific.

Glass wool.—Pittsburgh Plate Glass Co. SE 3 × 5—621 glass unbonded B.

Cigarette paper.—Commercial brand.

3,4 benzpyrene.—Eastman.

Methylcholanthrene.—Eastman.

Dibenz(ah)anthracene.—Eastman.

1,12 benzpyerylene.—Aldrich Chemical Co.

Initially, a Beckmann DU ultraviolet spectrophotometer was used, then a Beckmann DK-1 recording spectrophotometer for fluorescence and ultraviolet absorption work. For fluorescence (Burdett and Jones, 1947) the light housing backplate was modified to make the fluorescence sample the light source (Fig. 1). A small platform with a holder for a vial 1 cm. in diameter extended from the backplate, placing the vial at the same position normally occupied by the filament of the tungsten lamp. The ultraviolet exciting light was provided by a GE mercury vapor lamp (CH3) with appropriate transformer. This light passed through a hole in the backplate covered by a glass light filter (Corning 5860) which permitted light under 390m$\mu$ to enter the lamp housing. The mercury vapor lamp and transformer were mounted in a metal box provided with a hole adjacent to the lamp. A strip of aluminium foil was fitted to the back half of the lamp to serve as a crude reflector. The " energy " setting of the spectrophotometer and high sensitivity were used for fluorescence measurements.

### *Smoke Sampling*

Cigarettes and cigars were smoked in a smoke sampling apparatus designed by the research laboratory of The American Tobacco Company, Inc. and manufactured by Phipps and Bird, Inc. of Richmond, Virginia. In this apparatus each cigarette was smoked individually, one 3-second inhalation of 60 c.c. of air each minute. The smoke was drawn through acetone. Four cigarettes were smoked simulataneously.

Cigarette paper and tobacco were smouldered in a glass tube 1¼ inches in diameter and 12 inches long (Fig. 2). This tube was connected by means of a

---

EXPLANATION OF PLATE.

FIG. 1.—Beckmann spectrophotometer modified to make the fluorescence sample the light source.

FIG. 2.—Vertical glass tube 1¼ inches diameter and 12 inches long in which cigarette paper and tobacco were smouldered.

1

2

Cardon, Alvord, Rand and Hitchcock.

34/45 joint to the center neck of a 3-neck flask set in a water bath at room temperature. A piece of wire gauze was placed toward the bottom of the burning tube to prevent the ashes from falling into the flask. To the flask was fitted an adaptor leading to a glass tube tapered at the lower end containing a wad of glass wool 1 to 2 inches long. The tapered end of the condenser tube passed through a rubber stopper into a 200 c.c. filter flask. The filter flask was connected by vacuum tubing to a trap and then to a water aspirator.

*Chromatography*

Glass tubes $1\frac{1}{2}$ inches to 1 cm. in diameter and 2 to 3 feet long were used. Eluents were collected in narrow-mouth glass bottles 500 to 15 c.c. capacity.

### *Procedure*

*I. Cigarette Paper*

Cigarette paper was unwound from a commercial roll in 5 to 6 feet lengths. Thirty such lengths (about 30 g.) were wound together and torn into approximately 12 inch lengths. The burning tube was loaded with these wads. With the aspirator on full, the paper was lit and allowed to smoulder down to ashes. The burning front was periodically measured with a bare thermocouple junction and the temperature found to vary between 650 and 900° C. Temperatures to 850° C. were measured with an optical pyrometer. The air flow measured with a flowmeter averaged 5 1. per minute. Thirty minutes was the average time required for the complete burning of the 30 g. samples of paper and about 150 1. of air was passed. This corresponds to 30 1. of oxygen or 1·3 mols. or 7 mols. $0_2$/162 g. of cellulose. Theoretically, 6 mols of oxygen are required for the complete combustion of each 162 g. unit of cellulose so that an excess of oxygen was always used in these experiments.

Ten batches (300 g.) of paper were burned in an average day's experiment. Reddish tarry condensate accumulated on the sides of the flask, connecting tube, condenser, and especially in the glass wool plug. Liquid condensed in the glass wool and dropped into the filter flask. When 300 g. of paper had been burned, the apparatus was dismantled and rinsed down with 300 to 500 ml. of acetone (except for the burning tubes), which dissolved all of the condensed tars. The nonvolatile tars produced by the smouldering paper were determined by evaporating a sample of the acetone solution to dryness on a steam bath. Fifteen to 18 g. (5 to 6 per cent) of tars were obtained.

To the acetone solution in a separatory funnel, 500 ml. of cyclohexane was added, followed by 500 ml. of water. The aqueous-acetone water layer was drawn off and discarded. The cyclohexane solution was washed with two 500 ml. portions of water and dried over amhydrous calcium chloride. The dried cyclohexane solution was a sharp smelling, irritating reddish-brown liquid.

A 1 × 6 inch column of Harshaw chromatographic alumina, activated by heating in an oven at 130° C. for several hours, was prepared. The cyclohexane solution was added to the column and it was followed by pure benzene. The cyclohexane passed through the alumina, leaving all the colored material in a narrow band within 1 inch of the top of the column. If the alumina was insufficiently activated, it could be noted by the fact that the colored material spread through the column and came through in the cyclohexane eluents.

The addition of benzene started a yellow material down the column. When irradiated with ultraviolet light, this yellow material fluoresced a bright blue. Fifty milliliter eluents were collected and the fluorescence spectra of all fractions determined. The first benzene eluents were yellowish-green and fluoresced in one wide bright band with a peak at 440–450 m$\mu$ (Fig. 3). At the fourth or fifth benzene eluent, two peaks could be discerned at 410 to 412 and 432 to 435 (Fig. 4). The fluorescence spectrum of pure 3,4-benzpyrene in benzene solution has three major peaks at 410, 432, and 455 m$\mu$, with a smaller peak at 413 on the shoulder

FIG. 3.—Fluorescent spectra of eluents from the cigarette paper tar separation which do not contain 3,4-benzpyrene.

of the 410 peak. The 2 peak fluorescence noted in the benzene eluents of the chromatographic column was the first indication of the presence of 3,4-benzpyrene or other benzanthracene derivatives in the tar. Five to 6 eluents (about 300 ml.) had indications of the 2 peak fluorescence. Subsequent eluents fluoresced with a wide band background fluorescence. Much dark brown material remained on the column.

The eluents showing the 2 peak fluorescence were combined and evaporated to dryness in vacuo at 50 to 60° C. and the residue dissolved in 50 ml. of cyclohexane. This solution was added to a column, $\frac{3}{4} \times 4$ inches, of silica gel. A colored band remained within $\frac{1}{2}$ inch of the top of the absorbent layer. The column was developed with a solution of 10 per cent benzene in cyclohexane. Twenty-milliliter eluents were taken. The first benzene containing eluents fluoresced in bands with two prominent peaks at 410 and 432 m$\mu$ and a flat spot

FIG. 4.—Fluorescent spectra of eluents from the cigarette paper tar separation which contains 3,4-benzpyrene.

FIG. 5.—Fluorescent spectra. 1. Purified fraction of cigarette paper tar rich in 3,4-benzpyrene. 2. 3,4-Benzpyrene.

at 450 to 455 m$\mu$. The ultraviolet absorption curve showed a small peak at 385 and flat spots at 360 to 370 and 345 to 350 m$\mu$. Three hundred to 400 ml. were obtained.

The eluents with the 2 peak fluorescence were combined, evaporated to dryness and the residue dissolved in 25 ml. of cyclohexane. The cyclohexane solution was added to an alumina column 1 cm. in diameter and 2 inches long. The column was developed with 10 per cent benzene in cyclohexane. Ten milliliter eluents were taken. The ultraviolet absorption curves in eluents (15 to 25) showed definite peaks at 385 and 365 and suggestions of peaks at 347 and 405 m$\mu$.

The eluents with the suggestive ultraviolet absorption were combined, evaporated to dryness and taken up in 25 ml. of cyclohexane. This solution was added to a column of silica gel 1 cm. by 3 inches long. The column was developed with 5 per cent benzene in cyclohexane. The first benzene containing eluents had sharply defined peaks in the ultraviolet absorption spectra at 365, 385, and 405 with a flat spot at 345 to 350 m$\mu$. There was a flat shoulder at 380 m$\mu$ just below the peak at 385 m$\mu$. Two additional small columns of silica gel, developed with 3 and 1 per cent benzene in cyclohexane solutions, gave eluents with more clearly defined 3,4-benzpyrene absorption. Peaks at 333 and 317 suggest 1,2-benzpyrene is also present in these samples (Fig. 12). The fluorescence spectrum was very close to that of 3,4-benzpyrene (Fig. 5 and Fig. 6).

*Quantitative Estimation of the* 3,4 *Benzpyrene*

The eluents from the last chromatographic column were combined and an ultraviolet absorption curve run on the resulting solution (Fig. 12). An estimate was made of the 3,4-benzpyrene content of the solution using the method described in detail by Cooper (1954). In this method, linear background absorbance is asssumed over a small range under the most prominent peak of 3,4-benzpyrene (385 m$\mu$). A base line is drawn from a point (A) on the absorption curve at 375 m$\mu$ to another point (B) on the curve at 395 m$\mu$. A vertical line is dropped from the 385 peak (C) to its intersection (D) with the line. The absorbance at (D) is subtracted from the absorbance of the curve at 385 m$\mu$ (C). This is done for a solution of known concentration of 3,4-benzpyrene and for the solution whose concentration is to be determined; then,

Known absorbance/Unknown absorbance

= Concentration known/Concentration unknown.

In the sample shown, an eluent solution of 300 ml. from the chromatography of tars from 550 g. of cigarette paper gave the curve in Fig. 12. A solution of 0·001 per cent 3,4-benzpyrene in cyclohexane gave the other curve in Fig. 12.

| | 0·001% 3,4-benzpyrene in cyclohexane. | | Eluent solution. | |
|---|---|---|---|---|
| | C. | D. | C. | D. |
| % transmission | 10·8 | 55·2 | 43·0 | 62·1 |
| Absorbance | 0·967 | 0·258 | 0·367 | 0·207 |
| $A_C$—$A_D$ | 0·709 | | 0·160 | |

The percentage of 3,4-benzpyrene in the unknown solution was determined by substituting in the equation

$$\frac{0\cdot160}{0\cdot709} \times 10\ \mu g. \times 150 = 337\cdot2\ \mu g.$$

$$\frac{550}{0\cdot000337} = 1 \text{ part benzpyrene per 1,610,000 part of paper burned.}$$

To check the estimation method, 150 g. of paper were smouldered as above. The apparatus was washed with acetone. The acetone solution was divided in two equal portions. To one portion was added 5 ml. of a solution containing 10γ

FIG. 6.—Fluorescent spectra.

FIG. 7.—Fluorescent spectra.

of 3,4-benzpyrene per milliliter of cyclohexane or a total of 50 γ of 3,4-benzpyrene. The two portions of the acetone solution were chromatographically analyzed using the procedure outlined above. Ninty-two γ of 3,4-benzpyrene were obtained from one portion and 44 γ from the other. Thus, 48 γ of the added 50 were recovered.

*6-Iodo 3,4-Benzpyrene**

* See Tye, Graf and Horton (1955).

A solution obtained by chromatography of tars from cigarette paper smoke containing 97 $\gamma$ of 3,4-benzpyrene was evaporated to dryness. To a solution of the residue in 10 ml. of benzene was added half of a solution prepared by dissolving 1 g. of iodine in 10 ml. of benzene. The other half of the iodine solution was added to a $\frac{3}{4} \times 1\frac{1}{2}$ inch column of activated Alcoa F-20 alumina. The benzene solution of iodine and cigarette paper tar was then poured on to the column followed by pure benzene. The first benzene eluents containing iodine were shaken in a separatory funnel with a 5 per cent solution of sodium thiosulfate containing a crystal of potassium iodide. The decolorized benzene solution gave peaks in the ultraviolet absorption spectra at 402, 382, and 367 m$\mu$. The 402 peak was now the most prominent, whereas in the pre-iodination sample it was only a very small peak. The absorption of subsequent samples reverted to the 3,4 benzpyrene absorption ; evidently 6-iodo 3,4-benzpyrene is held more loosely by the alumina and thus comes off before 3,4-benzpyrene.

In order to enrich the 6-iodo 3,4-benzpyrene in the eluents, they were combined, evaporated to small volume and rechromatographed over a $\frac{1}{2} \times 3$ inch column of Alocoa F-20 alumina. The column was eluted with 10 per cent benzene in cyclohexane. A further separation of the iodo compound from unreacted 3,4 benzpyrene was effected. The first eluent contained an unidentified material with peak absorption at 330 m$\mu$, and was followed by eluents rich in the iodo compound, although still containing unidentified material with background absorption and some 3,4-benzpyrene (Fig. 8).

### *II. Tobacco*

Tobacco (from a popular brand of cigarettes), 120 g. in 30 g. batches, was smouldered in the same apparatus as used above for the cigarette paper (Fig. 2). After each 200 g. had been smouldered, the apparatus was dismantled and washed down with acetone. The acetone solutions were combined. The acetone solution (one liter) was diluted with one liter of cyclohexane followed by one liter of water. The aqueous-acetone layer was discarded. The cyclohexane solution was washed twice with water, with 600 ml. of 2 N HCl, and then with water until neutral.

The dried cyclohexane solution was passed through a $1\frac{1}{2} \times 10$ inch column of silica gel. The cyclohexane eluents were dark in color but were discarded after further chromatography failed to disclose the presence of any 3,4 benzpyrene. The origianl cyclohexane solution was followed on the column by 100 ml. of fresh cyclohexane and then by 800 ml. of benzene. The benzene solution fluoresced with high over-all fluorescence in the 400 to 450 m$\mu$ range, but indications of 3,4 benzpyrene fluorescence were not obtained because of masking by the background.

The benzene solution was evaporated to dryness in vacuo at 50–60° C. and the residue dissolved in 100 ml. of cyclohexane. This solution was added to a $\frac{3}{4} \times 4$ inch column of alumina and followed by pure benzene ; 20 ml. eluents were collected. Two hundred milliliters were obtained with 2 peak fluorescence at 410 to 420 and 435 to 445 m$\mu$ superimposed on a high background fluorescence.

These eluents were combined and evaporated to dryness as above. The residue was taken up in 50 ml. of cyclohexane and this solution added to a $\frac{3}{4} \times 5$ inch column of silica gel. The column was eluted by a solution of 10 per cent benzene in cyclohexane. The 2 peak fluorescence was improved but there were only slight breaks in the absorption curves at 385 m$\mu$.

The eluents showing 2 peak fluorescence were again combined, evaporated to dryness and the residue dissolved in 20 ml. of cyclohexane. This solution was chromatographed on alumina, 1 × 8 cm., eluting with 10 per cent benzene in cyclohexane. The first 200 ml. of eluents showed nothing. The next 50 to 100 ml. gave an absorption peak at 375 mμ probably due to anthracene. These were followed by eluents with peaks at 405, 385, and 365 mμ. Later eluents showed a peak at 383 mμ, possibly a derivative of 3,4-benzpyrene.

The eluents with the suggestive absorption were combined, evaporated to dryness in vacuo and the residue taken up in 25 ml. of cyclohexane. The next column was a small silica gel one, 1 × 8 cm.; the eluting solution was 10 per cent benzene in cyclohexane (10 ml. eluents). The absorption curves improved in eluents 5 through 12. A second silica gel column as above gave further improved absorption curves. The curve shown (Fig. 9) is the absorption of the residue from the appropriate eluents dissolved in 65 ml. of cyclohexane. Using this curve and the analytical method described above for cigarette paper, 152 μg. of 3,4-benzpyrene were obtained.

$$\frac{\log \frac{1}{0\cdot 30} - \log \frac{1}{0\cdot 44}}{\log \frac{1}{0\cdot 108} - \log \frac{1}{0\cdot 565}} \times 65 \text{ ml.} \times \frac{10\ \gamma}{\text{ml.}} = 152\gamma$$

$$\frac{1250}{0\cdot 000152} = 1 \text{ part benzpyrene per } 8{,}200{,}000 \text{ parts of tobacco burned.}$$

### *III. Cigarettes*

Cigarettes (three popular brands) were separately smoked in the cigarette smoke-sampling apparatus. Four hundred cigarettes were smoked in an experiment. The separation and identification technique for the tars was the same as that used on the tobacco tar. Estimates of 3,4-benzpyrene content were as follows : Brand A—40 μg.; Brand B—49 μg.; Brand C (filter tip, king size)—32 μg.

The estimation method was checked as in the case of the cigarette paper by adding 10 μg of 3,4-benzpyrene to one half of the acetone solution of tars from 380 cigarettes. Fifteen micrograms of 3,4-benzpyrene were obtained from one portion and 23·5 μg from the other ; the recovery was thus over 85 per cent of that added.

Indications of iodo-benzpyrene were obtained by conducting the catalytic iodination experiment on a purified fraction of the cigarette tars as described with the cigarette paper above.

### *IV. Cigars*

The smoking apparatus was that used for cigarettes, although modifications in the size of the tubes used as cigarette holders were necessary to accommodate the larger size cigars. Regular size and small size cigars were separately smoked. The separation and identification was that used on the tobacco described in the foregoing. The results were :

| | | |
|---|---|---|
| Brand A (regular size) | 82·5 | γ per 20 cigars (wt. of cigar 8 g.) |
| Brand A (small size) | 94·0 | γ per 49 cigars (wt. of cigar 2·75 g.) |
| Brand B (small size) | 10 | γ per 24 cigars (wt. of cigar 2·75 g.) |
| Brand C (regular size) | 25 | γ per 24 cigars (wt. of cigar 8·5 g.) |

In the eluents from Brand A containing 3,4-benzpyrene, a prominent peak is present at 340 mμ. This may be due to coronene.

FIG. 8.—UV absorption.

FIG. 9.—UV absorption.

FIG. 10.—UV absorption ; cigarette tars composite fraction.

DISCUSSION

The identification of 3,4 benzpyrene in the tars from the smoke of cigarettes, tobacco, and cigarette paper is based on four considerations.

1. The fluorescence spectrum. The original crude spectra of the first investigators in this field could hardly be considered identification, since they reported 3 bands in the wavelength region, 4000 to 4500 Å (Cook, Hewett and Hieger, 1933 ; Kennaway and Hieger, 1930). The spectra recorded with a wide slit show only 3 bands, but even so a difference is readily apparent between 3,4-benzpyrene, 1,2,5,6 dibenzanthracene, and 10 methylcholanthrene (Fig. 7). The narrow slit fluorescence spectrum discloses a still more complex spectrum for 3,4-benzpyrene (Fig. 6) ; 4 distinct peaks at 406, 408, 430, and 450 with fine irregularities in the region 410 to 420 m$\mu$. This fine structure fluorescence spectrum of 3,4-benzpyrene was first noted by Bernard Muel and Michel Hubert-Habart at The Institute du Radium in Paris, France. The apparatus for fluorescent spectra used in that laboratory was designed by the director of the laboratory, R. Latarjet, and is capable of high sensitivity and high dispersion. Nevertheless, much more work is necessary before fluorescence itself can be considered final identification, since carefully determined spectra of many 3,4-benzpyrene-like compounds must yet be done.

2. Ultraviolet absorption spectrum. The identification using absorption spectra is more certain than is fluorescence alone. Five distinct peaks can be seen, at 405, 385, 380, 365, 347, with a discontinuity at 395 m$\mu$. The spectra of only two rare polynuclear hydrocarbons of many that have been reported are closely enough aligned to that of 3,4 benzpyrene to raise serious doubts of the identity (9-methylanthracene and 1 : 12-benzperylene) (Friedel and Orchin, 1951). Fluorescence eliminates the latter as its fluorescence spectrum is completely different from that of 3,4-benzpyrene (Fig. 11).*

3. The indication of the presence of 6-iodo 3,4-benzpyrene in iodinated purified fractions of the tars (Tye, Graf and Horton, 1955).

4. The recovery of added quantities of pure 3,4-benzpyrene in the same eluents by the identical procedures as with the substance being investigated.

Further, there is indication of the presence of 1,2-benzpyrene (absorption peaks at 333 and 317 m$\mu$) in the same eluents with the 3,4-benzpyrene, as would be expected. This is especially clear and pronounced in the excellent absorption curve obtained by Lefemine at the Miami Cancer Institute, on a highly purified fraction of cigarette paper tar.

The weight of a cigarette is about 1 g. The weight of the paper is 0·04 g. Assuming the paper and tobacco produce the same quantitites of 3,4-benzpyrene when smoked in a cigarette as they do when smouldered separately in the apparatus described here, the paper of a cigarette would produce 0·042 and the tobacco 0·122 $\gamma$. The total per cigarette is then 0·162 or for 400 cigarettes, 64·2 $\gamma$. This is in line with the results obtained with cigarettes smoked in the smoking machine (40 to 49·2), considering that $\frac{1}{4}$ to $\frac{1}{5}$ of the cigarettes are discarded as butts in the latter experiment.

* Since the preparation of this paper, a sample of 9-methylanthracene was obtained. The fluorescence of this compound is also very much different from that of 3,4-benzpyrene and can be used to differentiate between the two compounds.

FIG. 11.—Fluorescence.

FIG. 12.—UV absorption.

The variation in 3,4-benzpyrene formation in the smoke of the different brands of cigars suggests the high value in the one brand may be a product of an additive used in this tobacco. The other brands show the expected similarity to tobacco.

The authors are grateful to Dr. M. S. Newman of Ohio State University for his advice and help and to Mr. J. Ruggiero for the preparation of the photographs and figures.

### REFERENCES

Burdett, R. A. and Jones, L. C., Jr.—(1947) *J. opt. Soc. Amer.*, **37**, 7, 554.
Cook, J., Hewett, C. and Hieger, I.—(1933) *J. chem. Soc.*, 395.
Cooper, R. L. (1954) *Analyst*, **79**, 573.
*Idem* and Lindsey, A. J. (1954) *Chem. & Ind.* (*Rev.*), 1260.
*Iidem* and Waller, R. E.—(1954) *Ibid.*, 1418.
Doll, R. and Hill, A. B.—(1950) *Brit. med. J.*, **2**, 739.
Friedel, Robert A. and Orchin, Milton—(1951) 'Ultraviolet Spectra of Aromatic Compounds.' New York (John Wiley & Sons, Inc.).
Hammond, E. Cuyler and Horn, Daniel—(1954) *J. Amer. med. Ass.*, **155**, 1316.
Kennaway, E. L. and Hieger, I. (1930) *Brit. med. J.*, **1**, 1044.
Tye, Russel, Graf, Mary Jane and Horton, A. Wesley—(1955) *Analyt. Chem.*, **27**, 248.
Wynder, E. L. and Graham, E. A.—(1950) *J. Amer. med. Ass.* **143**, 329.
*Iidem* and Croninger, A. B.—(1953) *Cancer Res.*, **13**, 855.